# Assessing the validity of and factors that influence accurate self-reporting of HIV status after testing: a population-based study

**DOI:** 10.1097/QAD.0000000000002513

**Published:** 2020-01-30

**Authors:** Steady J.D. Chasimpha, Estelle M. Mclean, Albert Dube, Valerie McCormack, Isabel dos-Santos-Silva, Judith R. Glynn

**Affiliations:** a Faculty of Epidemiology and Population Health, London School of Hygiene & Tropical Medicine, London, UK; b Malawi Epidemiology and Intervention Research Unit, Karonga, Malawi; c International Agency for Research on Cancer (IARC), Lyon, France.

**Keywords:** HIV status, Malawi, self-report, sub-Saharan Africa, validity

## Abstract

To assess the validity of self-reported HIV status, and investigate factors that influence accurate reporting of HIV-positive status, in a population tested and informed of their HIV test result.

**Design:**

Prospective cohort study.

**Methods:**

We compared self-reported HIV status with biomarker-confirmed HIV test status among participants of Karonga Health and Demographic Surveillance Site in rural northern Malawi. We linked information on HIV test results to subsequent self-reported HIV status, and calculated sensitivity, specificity, positive predictive value and negative predictive value for self-reported HIV status (considered as a diagnostic test). We used Poisson regression with robust variance estimators to examine predictors of accurate self-reporting of HIV-positive status.

**Results:**

Among 17 445 adults who tested for HIV, were recorded as having received their HIV test results, and had a subsequent self-reported HIV status between 2007 and 2018: positive predictive value of self-reported HIV status was 98.0% (95% confidence interval: 97.3–98.7); negative predictive value was 98.3 (98.1–98.5); sensitivity was 86.1% (84.5–87.7); and specificity was 99.8% (99.7–99.9). Among true HIV-positive people, those who were younger, interviewed in community settings, and had tested for HIV longer ago were more likely to misreport their HIV-positive status.

**Conclusion:**

In this setting, self-report provides good estimates of test-detected HIV prevalence, suggesting that it can be used when HIV test results are not available. Despite frequent HIV testing, younger people and those interviewed in community settings were less likely to accurately report their HIV-positive status. More research on barriers to self-reporting of HIV status is needed in these subgroups.

## Introduction

With the wide availability of HIV-testing, many people already know their HIV status and may be reluctant to retest for research studies. Self-reported HIV status may be used, but participants may misreport. This may be for fear of stigma and discrimination [1] or when they feel that there are incentives and/or extra support services associated with a particular HIV status [2]. For example, reporting to be HIV-positive anticipating customized care or HIV-negative to be recruited into studies.

Despite self-report being a useful source of HIV data, its validity has not been well characterized. Most prior studies on self-reported HIV status in sub-Saharan Africa (SSA) focused on people's perception of their likelihood of HIV infection and actual HIV test results [2,3]. There are few studies that compared self-reported HIV data against HIV test results in SSA. In a South African study in older adults, concordance between self-reported HIV status and serology was very high among seronegative people (specificity 98.4%), but low among seropositive people (sensitivity 66.2%) [4]. As HIV self-reports preceded the HIV test, the study included individuals who did not know their HIV status. However, restricting to only those who knew their status did not improve sensitivity estimates. Among people who reported knowing their HIV status in demographic surveys in Malawi (2010) and Uganda (2011), agreement between self-reported HIV status and test-detected HIV status was even lower (26.1–44.2%) [5]. In the 2012 Kenya AIDS Indicator survey, sensitivity for self-reported HIV status was 47% [6].

As access to HIV testing and counselling services continue to expand, more people will become aware of their HIV status. This poses challenges on willingness to participate in HIV testing especially for individuals who already know their HIV-positive status [7]. Here, we assess the validity of self-reported HIV status among adults (aged 15 and above) who had been tested and informed of their test results, using systematically collected prospective data from Karonga Health and Demographic Surveillance Site (HDSS) in rural northern Malawi. We compare performance of self-reported HIV status between community and clinic settings. We focus on sensitivity rather than specificity, to examine factors that influence accurate reporting of an HIV-positive status.

## Methods

### Study setting and population

We used population-based cohort study data on HIV test results and subsequent HIV self-reports collected by the Malawi Epidemiology and Intervention Research Unit (MEIRU, formerly known as the Karonga Prevention Study). Apart from conducting population-based epidemiological studies, MEIRU also runs the Karonga HDSS [8]. The HDSS was established in 2002 in rural northern district of Malawi, routinely collecting information on births, deaths (monthly) and migrations (annually). Regular community-wide surveys are conducted to capture information on socio-economic status, monitor HIV-infection patterns and evaluate impact of interventions carried out in the area. There are now over 40 000 people in the HDSS, most of whom are rural subsistence farmers, fishermen and small traders [9].

### HIV data

Following a sample serosurvey completed between 2005 and 2006 [7,10], HIV data in the HDSS are available mainly through regular population-wide house-to-house cross-sectional serosurveys. By 2011, a total of four such surveys had been conducted using different types of rapid HIV tests [10]. In addition, HIV testing is offered in clinics and research studies [7]. Self-reported data on previous HIV testing, approximate date, and result of most recent HIV test are collected at the time of HIV testing. Consenting participants may choose not to be informed of their HIV test results. HIV testing is also available from service providers within and outside the HDSS.

We used the participant unique identification number and dates to link all HIV test results to corresponding data on subsequent self-reported HIV status, and created record pairs between the HIV test and self-reported HIV status. As such we had multiple records of HIV test results and subsequent self-reports per individual. For simplicity, and to get the most contemporaneous results, we analysed the most recent pair of an HIV test and its subsequent self-reported HIV status in individuals who chose to receive, and were given their results.

### Socio-economic and demographic data

Socio-economic and demographic data in Karonga HDSS are usually updated during the annual HDSS surveys. These include marital status, level of education, and occupation. Using occupation and reliability of income, we created an employment score as an indicator of socio-economic status, in which low is the least skilled/reliable (e.g. piece work) and high is the most skilled/reliable (e.g. government worker paid monthly). The medium category consists of predominantly self-employed subsistence farmers. We assessed area of residence using distance between participants’ residence and the tarmac road [10,11].

### Statistical analysis

We restricted the analysis to adults aged 15 years and above, and calculated true HIV prevalence, as assessed by the rapid HIV tests, in each category of self-reported HIV status (negative, positive, don’t know, refuse to disclose and never tested) according to whether participants received their HIV test result. Using Pearson's chi-squared test, we assessed distribution of participants’ socio-demographic characteristics by self-reported HIV status, recorded as positive, negative and unknown (the latter included individuals who reported ‘don’t know,’ ‘refuse to disclose’ and ‘never tested’). We then assessed performance of self-reported HIV status against serological HIV test result obtained from the rapid HIV tests – regarded here as the ‘gold-standard’.

Restricting to individuals who were recorded as having received their test results, and who self-reported being either HIV-positive or HIV-negative (i.e. excluding those who self-reported HIV-unknown status) we estimated [with their 95% confidence intervals (CIs)]:

(1) Sensitivity: Probability of self-reporting HIV-positive among those who tested HIV-positive.
(2) Specificity: Probability of self-reporting HIV-negative among those who tested HIV-negative.
(3) Positive predictive value (PPV): Probability of testing HIV-positive among those who self-reported HIV-positive.
(4) Negative predictive value (NPV): Probability of testing HIV-negative among those who self-reported HIV negative [12,13].

Changes in sensitivity and specificity by time between the HIV test and the self-report were assessed.

We also examined factors associated with the accuracy of self-reported HIV status. Only serologically HIV-positive individuals who were known to have received their HIV test results were included in this analysis. The outcome was binary (yes or no) for accurately reporting being HIV-positive. We used Pearson's chi-squared test for equality of proportions between populations, to identify variables associated with accurate reporting of HIV-positive status at *P* value less than 0.2, for assessment in multivariable models. Because of a high proportion reporting HIV-positive, we estimated prevalence ratios rather than odds ratios as a measure of association, using modified Poisson regression models (with robust variance estimators). Robust variance estimators were used to correct for wider CIs that would be observed in regular Poisson models [14,15]. The basic model included age, sex and calendar year of HIV self-report *a priori*. Other variables were added one at a time to choose a parsimonious yet best fitting model.

For those who had more than one pair of self-reports followed by test results, we compared the first pair with the most recent pair to see if there were changes in self-reporting (whether accuracy improved) in individuals who had retested. We used Stata 16 (StataCorp, College Station, Texas, USA) and R-software (R Development Core Team 2019, Vienna, Austria) for analyses and graphics, respectively.

### Ethics

Ethical approval for demographic surveillance and HIV studies in Karonga HDSS area were obtained from the Malawi National Health Sciences Research Committee (approval #s NHSRC/01/38 and 419), and the research ethics committee of London School of Hygiene & Tropical Medicine (# 5081). For this analysis, additional approval was obtained from the London School of Hygiene & Tropical Medicine ethics committee (#16495).

## Results

Between 2007 and 2018, 17 856 adults were tested for HIV and had a subsequent self-reported HIV status. Of these, 10 148 (56.8%) were women and 7709 (43.2%) were men. Median age was 31.5 years (interquartile range: 22.7–44.2). Nearly all participants [17 445 (97.7%)] received their HIV test results, with just 145 (0.8%) individuals choosing not to know their HIV test results, and 266 (1.5%) with missing data on receipt of HIV test results. Overall, there were 2046 (11.5%) HIV-infected people. HIV prevalence was higher among women (12.1%) than men (10.6%). Self-reported data on current antiretroviral therapy (ART) use was available for 1423 (69.5%) of all individuals with an HIV-positive test. Of these, 1171 (82.3%) reported to be on ART. ART data were missing/unknown for 623 (30.5%) of all HIV-positive individuals.

**Fig. 1:**
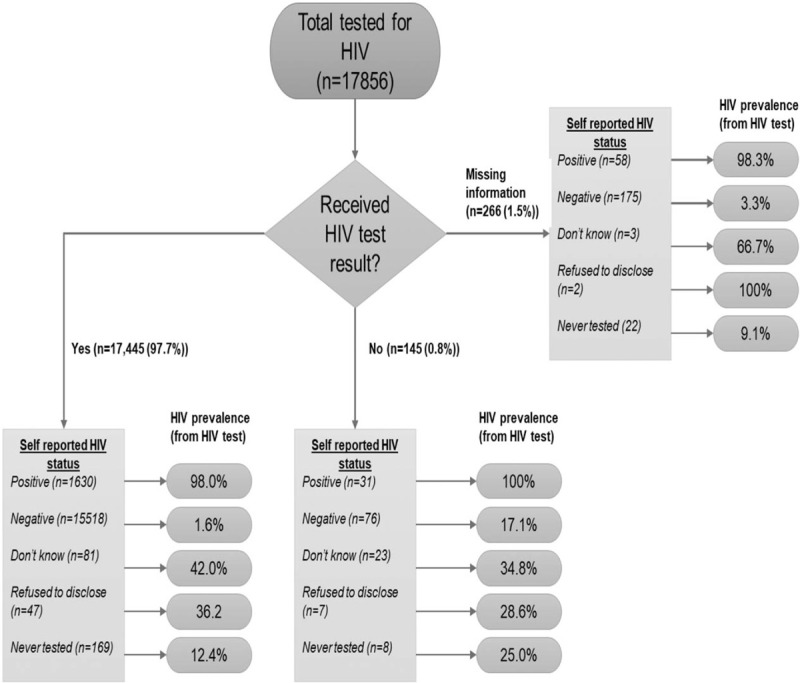
HIV prevalence and self-reported HIV status according to whether participants received their HIV test results.

Among those who had received their HIV test results: true HIV prevalence was 98.0% in individuals who self-reported as HIV-positive (i.e. the PPV); 1.6% among self-reported HIV-negatives; 42.0% among those self-reporting not knowing; 36.2% among those refusing to disclose; and 12.4% among those self-reporting to have never been tested (Fig. 1).

### Self-reported HIV prevalence

Table 1 shows participant characteristics by self-reported HIV status. Overall self-reported HIV prevalence (excluding those who reported ‘unknown’) was 9.6%, whereas the serological HIV prevalence in the same individuals was 11.5%.

**Table 1:**
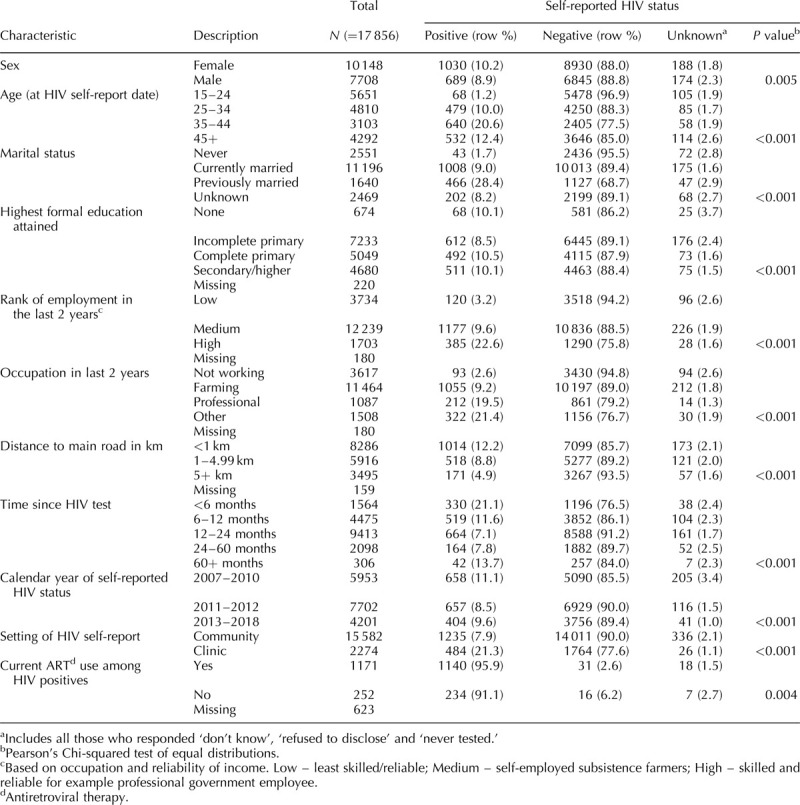
Participants’ characteristics in Karonga Health and Demographic Surveillance Site, and their association with self-reported HIV status.

### Performance of self-reported HIV status among those who were recorded as having received their results

The joint distribution of self-reported and true HIV status among the 17 148 participants who received their results and had valid self-reported HIV status (i.e. excluding those self-reporting ‘unknown’), was 89.0% true negatives, 9.3% true positives, 0.2% false positives and 1.5% false negatives that is, discordant pairs were predominantly of HIV-positive individuals reporting as HIV-negative, whereas it was rare for an HIV-negative individual to report being HIV-positive.

The overall sensitivity for self-reported HIV status was 86.4% (95% CI: 84.8–88.0%) and specificity was 99.8% (99.7–99.9%). PPV and NPV were also high 98.0% (97.3–98.7%) and 98.4% (98.2–98.6%), respectively, (Table 2). The estimates were similar when all participants were considered (i.e. regardless of whether individuals were recorded to have received their HIV test result): sensitivity was 86.2% (84.6–87.7) and specificity was 99.8 (99.7–99.9). PPV and NPV were 98.1% (97.3–98.7) and 98.3% (98.1–98.5), respectively.

**Table 2:**
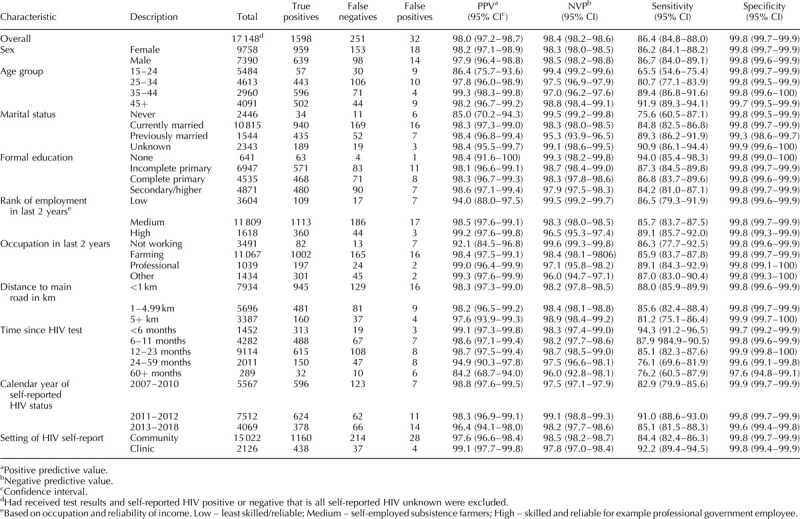
Performance of HIV self-report against serological HIV test among 17 148 individuals who received their HIV test results.

Both sensitivity and PPV increased with age: sensitivity was 65.5% in 15–24 year olds compared with 91.9% in those aged 45 years and over. Individuals who had never married had lower sensitivity of self-reported HIV status (75.6%; 60.5–87.1%) than those currently (84.8%; 82.5–86.6%) or previously married (89.3%; 86.2–91.9%). Sensitivity and PPV were slightly higher for self-reported HIV status conducted in clinic settings [92.2% (89.3–94.3%) and 99.1% (97.7–99.8%, respectively] compared with community settings [84.4% (82.0–85.9%) and 97.6% (96.6–98.4%), respectively; Table 2].

The longer the duration between HIV testing and self-reported HIV interview, the lower the sensitivity and PPV for self-reported HIV status. Sensitivity was 94.3% (91.2–96.5%) among those reporting within six months of HIV testing compared with 76.1% (69.6–81.9) among those self-reporting 2–4 years after the test. Overall, specificity was similar and very high (>99%) across all levels of socio-demographic factors except for those with an interval of 60 months or more (97.6%).

**Fig. 2:**
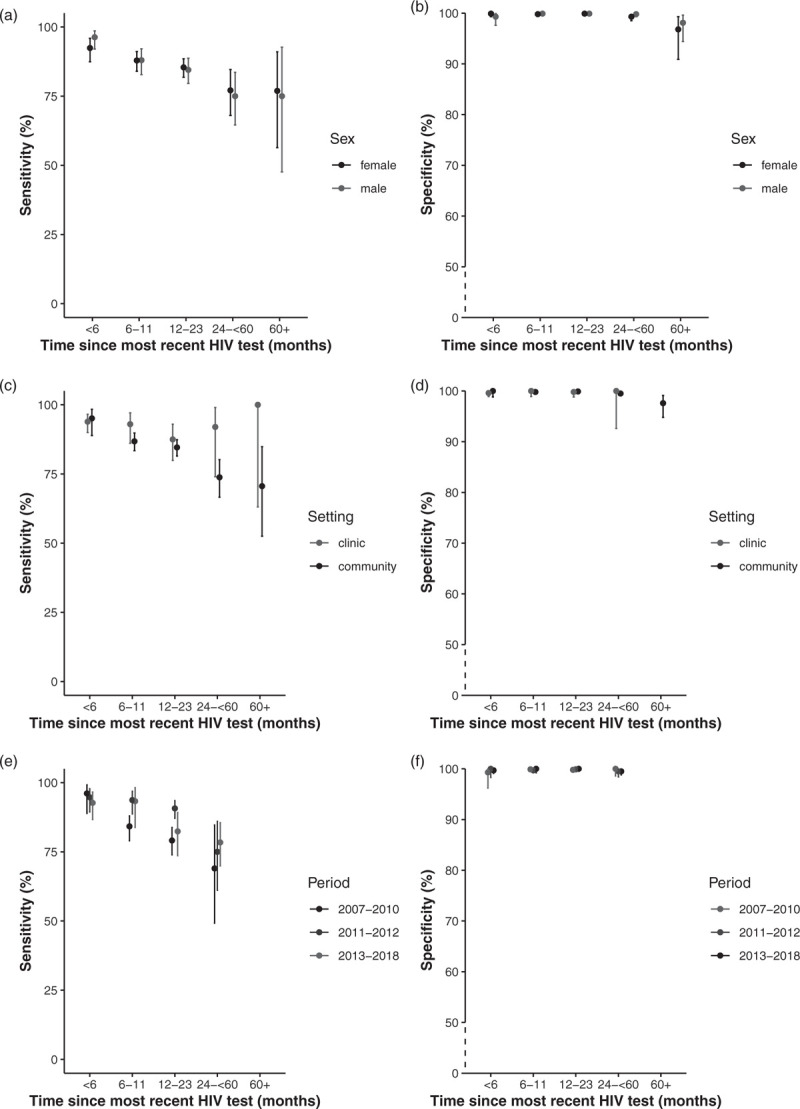
Sensitivity and specificity of self-reported HIV by time since most recent HIV test, stratified by sex (a and b), and type of setting (c and d) and calendar year of self-report (e and f).

*Note:* Estimates for clinic 60+ months in (d) not displayed because it was based on just nine records (specificity = 100%, 95% confidence interval: 2.5–100).

Figure 2 shows plots of sensitivity and specificity of self-reported HIV status and time since most recent HIV test by sex, setting and calendar period. For the decline in sensitivity with increasing time since most recent HIV test, the pattern was similar for males and females, but differed by setting, with sensitivity in clinic settings remaining higher than in community settings.

Sensitivity decreased with increasing time since most recent HIV across all calendar periods (in which self-reported HIV status was collected), but was markedly lower in the earlier period (2007–2010) than in latter periods (2011–2012 and 2013–2018). However, there were no self-reports beyond 5+ years in earlier periods. Overall, specificity remained high (near 100%) regardless of length of the time interval between HIV testing and self-reporting (Fig. 2).

There were 8076 individuals with at least two pairs of self-report and HIV test result. This included 47 (0.6%) individuals who seroconverted between tests, of whom 26 (55.3%) reported their new HIV-positive status accurately (Supplementary Table 1). Excluding those who seroconverted, self-reported HIV status was consistent (i.e. correct in both) in 7939 (98.8%) individuals. Among 446 HIV-positive individuals; 375 (84.1%) consistently accurately reported their HIV-positive status. Self-reporting HIV-positive status improved (i.e. correct in last but not first test) in 44 (9.9%); worsened in 10 (2.2%); and consistently incorrect in 17 (3.8%) individuals (Supplementary Table 2).

### Predictors of accurate self-reported HIV-positive status

Among 1849 HIV-infected individuals who knew their HIV status, 1598 (86.4%) accurately self-reported their HIV-positive status (i.e. sensitivity; Table 2). In multivariable analyses, accurate self-reporting of HIV-positive status did not statistically significantly differ by sex, highest education attained, rank of employment/occupation or distance to main road (Table 3). Individuals who were younger were less likely to accurately report their HIV-positive status than older adults (chi-square test for trend: *P* < 0.0001). When compared with 45+ years age group, the adjusted prevalence ratios was 0.71 (95% CI: 0.61–0.83) among 15–24 years old and 0.87 (0.83–0.91) in 25–34 age group.

**Table 3:**
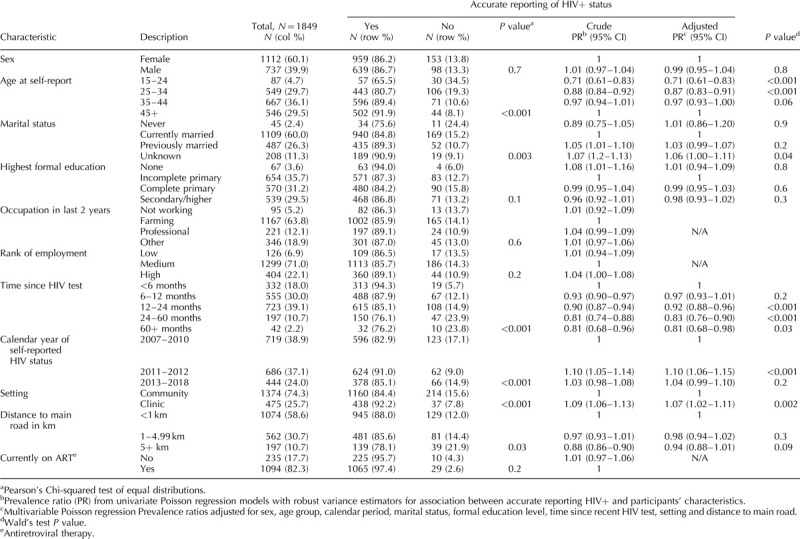
Predictors of accurate reporting of HIV-positive status among HIV-infected individuals who received their HIV test results.

Shorter periods between HIV testing and self-reporting HIV status, were associated with correctly reporting being HIV-positive (chi-square test for trend: *P* = 0.004). HIV-infected individuals interviewed in clinic settings were more likely to accurately report their positive status than those in community settings (adjusted prevalence ratios: 1.07; 95% CI: 1.02–1.11) (Table 3).

## Discussion

Understanding accuracy and determinants of correct reporting of HIV status is essential for HIV programs and epidemiological studies that may rely on self-reported HIV status data. In a large population-based study in rural northern Malawi of community members who had received an HIV test and been informed of the results, we found very high specificity, and high sensitivity for self-reported HIV status. We did find a small proportion (0.2%) of false positives – individuals self-reporting to be HIV-positive whose last known HIV result was negative, suggesting that our ‘gold standard’ HIV test database was extremely good but not perfect. These false positives were not surprising as HIV testing is available outside the study setting and such individuals may have tested positive elsewhere, a result not captured by the study. False positives were more common with large gaps between HIV testing and self-report, consistent with seroconversion after our last recorded test. Specificity and NPV were similar across different socio-demographic factors.

We observed higher PPV and sensitivity in older age groups compared with young ones. As shown in other studies, older people are more likely than young adults to disclose their HIV status due to having steady sexual relationships and sense of responsibility [16,17]. Individuals who had never married had lower sensitivity (75.6%: 60.5–87.1) and PPV (85.0%: 70.2–94.3) compared with those who were currently (PPV 98.3%) or previously married (98.4%). However, the association between accurately self-reporting an HIV-positive status and marital status was lost when marital status was adjusted for age and other factors (Table 3).

Similar to other studies [4] sensitivity and PPV decreased with increasing time interval between HIV testing and self-reported HIV status (Fig. 2). It might be expected that individuals recently tested would live in denial (lower PPV), and gain acceptance over time (higher PPV), but this was not supported by the data, and we were not necessarily looking at time since first HIV-positive test. It is possible that individuals with longer time intervals were more likely not to believe the test results especially if they remained healthy, leading to misreporting their status. Being on ART has been shown to be associated with high sensitivity for self-reported HIV status [4]. We found no evidence for an association between current ART use and accurately reporting HIV-positive status, partly due to the substantial amount of missing data (30%) on ART use. Accuracy in self-reporting remained consistent for most individuals when we compared the first and last set of HIV test result and subsequent self-reported HIV status. However, there was evidence, albeit based on small numbers, that individuals who seroconverted between tests were more likely to misreport their HIV-positive status perhaps because they were still living in denial.

Accuracy of self-reported HIV status also depends on the context and setting in which information is being reported and perceived benefit or harm of disclosure. Our estimates of sensitivity and PPV were higher (92.1, 99.1%, respectively) in clinic settings compared with community settings (84.1, 97.7%) (Table 2). As patients, they may feel obligated to tell the truth for healthcare providers to take necessary precaution or in anticipation of optimized care. Unlike in community settings in which privacy issues (presence of family/friends nearby) and mistrust of fieldworkers/interviewers are likely to influence misreporting.

Sensitivity for self-reported HIV status increased from 83% during 2007–2010 to 91% (2011–2012) before dropping to 85% (2013–2018) (Table 2). The 2007–2010 period was the time annual HIV serosurveys were being introduced. By 2011, a total of four such surveys had been completed. Therefore, people were more likely to have had multiple tests during the 2011–2012 period. Also they were likely to feel more comfortable talking to study fieldworkers and therefore, more willing to share their HIV status than in the earlier period. As such, the observed higher sensitivity during 2011–2012 than in 2007–2010 are expected. The 2013–2018 period saw major changes in national HIV/ART guidelines including expansion of Option B+ [18,19] to all antenatal care clinics in Malawi in 2013 [20,21], and adoption of the 2015 WHO guidelines [22] on universal ‘test and treat’ in 2016 [23–25]. It might be expected that more people would be aware of their HIV status during this period resulting in higher sensitivity for HIV status. However, sensitivity was lower in 2013–2018 than in 2011–2012, probably because HIV serosurveys were no longer conducted in the HDSS during this period.

Our estimates of sensitivity are higher than those in prior studies from South Africa, Malawi and Kenya, which ranged from 26% in Malawi to 51.2% in South Africa [4,5]. This may be because our analysis was restricted to individuals who had been informed of their HIV results, in a population that had been frequently tested. While in prior studies, self-reporting preceded the HIV test; sensitivity estimates were based on responses to the question ‘have you ever tested positive for HIV?’ thereby including individuals who did not know their HIV status. However, in one of the studies, sensitivity and PPV remained essentially the same in a subset of participants that reported knowing their status [4].

Our study is large and based on population-based data spanning over 10 years. We explored predictors of accurate self-reporting of HIV-positive status in SSA, including assessing its performance in different settings (clinic vs. community). A limitation to this study is that the findings may not be easily generalizable to other settings. The regular HIV serosurveys and other research studies conducted in the HDSS mean that participants are exposed to frequent HIV testing and are aware of their HIV status more than other settings. This may be the reason for the observed higher predictive values (even in community settings) compared with prior studies in the region. However, more people in SSA are now aware of their HIV status. In 2018, an estimated 85% (75–95) of PLWH in East and Southern Africa knew their status, higher than the estimated global 79% [26]. For 90% in the UNAIDS 90–90–90 [27] to be achieved, frequent HIV testing and retesting is required. This will help improve sensitivity of self-reported HIV – an important resource for HIV management programs and epidemiological studies.

In conclusion, the validity of self-reported HIV status was high. We observed very high specificity and NPV. Our estimates of sensitivity and PPV were higher than those reported in other studies in SSA. Being younger, interviewed in community settings and having longer duration since most recent HIV test was associated with less accurate reporting of HIV-positive status. Our findings confirm self-reported HIV status, and especially self-reported positive status, as a useful measure of HIV status when test data are unavailable.

## Acknowledgements

J.R.G. conceived the study and contributed to design of the original data collection. A.D. contributed to data collection. J.R.G, S.C. and E.M. designed the analysis. S.C. led the statistical analysis, with contributions from J.R.G., I.D.S.S, E.M., and V.M. The article was written by S.C., with contributions from all other authors. All authors have read and approved the submitted version of the article.

### Conflicts of interest

All studies contributing the data for current analysis were funded by the Wellcome Trust (Grant ref # 098610/Z/12/Z). S.C. is a Commonwealth scholar funded by the UK government.

Where authors are identified as personnel of the International Agency for Research on Cancer/WHO, the authors alone are responsible for the views expressed in this article and they do not necessarily represent the decisions, policy or views of the International Agency for Research on Cancer/WHO.

## Supplementary Material

Supplemental Digital Content
