# Supplementary material for: Assessing the validity of and factors that influence accurate self-reporting of HIV status after testing: a population-based study
Source: AIDS. 2020 Jan 30;34(6):931–41. doi: 10.1097/QAD.0000000000002513 (PMC7553190; doi:10.1097/QAD.0000000000002513)
Supplement: Supplemental Digital Content [file aids-34-931-s001.docx]

Supplementary Table 1: Comparison of self-reporting of HIV status between the first and last set of HIV test result and subsequent self-reported HIV status among individuals who seroconverted.

|  | All (N=47) | |
| --- | --- | --- |
| Change in self-reporting | N | % |
| Correct in both | 26 | 55.3 |
| Correct in first not last | 20 | 42.6 |
| Correct in last not first | 0 | 0.0 |
| Incorrect in both | 1 | 2.1 |

Supplementary Table 2: Comparison of self-reporting of HIV status between the first and last set of HIV test result and subsequent self-reported HIV status (excluding individuals who seroconverted).

|  | **HIV test result** | |  |
| --- | --- | --- | --- |
|  | **Negative** | **Positive** | **Total** |
| **Change in self-reporting** | **N (%)** | **N (%)** | **N (%)** |
| Correct in both | 7564 (99.8) | 375 (84.1) | 7939 (98.8) |
| Correct in first not last | 12 (0.2) | 10 (2.2) | 22 (0.3) |
| Correct in last not first | 7 (0.09) | 44 (9.9) | 51 (0.7) |
| Incorrect in both | 0 (0.0) | 17 (3.8) | 17 (0.2) |
| **TOTAL** | **7583 (100)** | **446 (100)** | **8029 (100)** |
